# Proton Magnetic Resonance Spectroscopy for the Early Diagnosis of Parkinson Disease in the Substantia Nigra and Globus Pallidus: A Meta-Analysis With Trial Sequential Analysis

**DOI:** 10.3389/fneur.2022.838230

**Published:** 2022-06-16

**Authors:** Wenbin Gu, Chen He, Juping Chen, Junchen Li

**Affiliations:** ^1^Department of Radiology, Changshu Hospital Affiliated to Nanjing University of Chinese Medicine, Suzhou, China; ^2^Department of Radiology, Nantong Rich Hospital, Nantong, China

**Keywords:** globus pallidus, meta-analysis, Parkinson disease, proton magnetic resonance spectroscopy, substantia nigra, trial sequential analysis

## Abstract

This study aimed to investigate the metabolic changes in globus pallidus (GP) and substantia nigra (SN) during the early stage of Parkinson disease (PD) using magnetic resonance spectroscopy (MRS). PubMed, Embase, Web of Science, and Chinese National Knowledge Infrastructure were searched till November 2018. Eligible trials comparing early metabolic changes in GP and SN in patients with PD vs. controls were included. The mean differences with 95% confidence intervals were estimated with either fixed- or random-effects models using Review Manager 5.3 software. Trial sequential analysis was performed using TSA 0.9.5.10 beta software. Finally, 16 studies were selected from the search. Overall, the *N*-acetyl aspartate-to-creatine ratio showed a significant difference between patients with early-stage PD and healthy controls. The overall heterogeneity was *P* < 0.00001, *I*^2^ = 94% in GP and *P* = 0.0002, *I*^2^ = 74% in SN. The results revealed that MRS could be a more sensitive imaging biomarker in the diagnosis of early-stage PD.

**Systematic Review Registration:**

https://www.crd.york.ac.uk/prospero/display_record.php?RecordID=125731, registration number: CRD42019125731.

## Introduction

Parkinson disease (PD) is a degenerative neurologic disease characterized by the loss of dopaminergic neurons in the cerebral gray matter nuclei such as globus pallidus (GP) and substantia nigra (SN) (1). To date, it is well-accepted that the loss of neuromelanin in the SN plays an important role in the pathology of PD, with which the different functional imaging methods have been used to detect the changes (2). The common neuropathological processes in PD can be evaluated by decreased *N*-acetyl aspartate (NAA) concentrations and changes in choline (Cho) and creatine (Cr) levels. NAA in the cerebral regions of patients with PD acts as an indirect marker of the integrity of neurons, possibly indicating a neuronal loss. Also, creatine (Cr) acts as a marker for energy metabolism, indicating phospholipid membrane synthesis (3).

In recent years, reliable proton magnetic resonance spectroscopy (^1^H MRS) markers of neurodegenerative changes in PD patients may act as a clinical auxiliary diagnosis but are not yet to be developed (4). Furthermore, a significant correlation has been found between the decrease in the NA-to-Cr ratio (NAA/Cr) and the global cognitive decline independent of motor impairment (5). These markers may be found in the cerebral regions of patients with PD, including the SN, corpus striatum (it is generally defined as basal ganglia including lentiform nucleus made up of the putamen and GP, caudate, and thalamus), motor cortex, cingulate, and prefrontal associated cortices (6). These structures, especially in GP and SN, form a set of parallel neuronal loops that serve as motor, cognitive, and affective functions often impaired in PD. Therefore, morphological changes and changes in energy metabolism are observed in patients with PD because of neuronal loss in GP and SN (7).

However, the decrease in the NAA/Cr in GP and SN in the early diagnosis of PD prior to MR changes and even before any clinical symptoms is still controversial (8–11). In 2013, Baglieri et al. (12) conducted a systematic review of eight trials. Two studies (13, 14) demonstrated no significant difference in the NAA/Cr in the SN of patients with PD. Another study (15) showed a reduced NAA/Cr in the putamen, correlating well with the severity of PD. Another systematic review in 2016 (16) included 14 trials about MRS and showed a decrease in the NAA/Cr in the left symptomatic side of SN performed in 1.5T MR (17) and in 3.0T MR, a decrease in the NAA/Cr in the rostral SN regions, and an increase in the NAA/Cr in the caudal SN regions (18). Therefore, a meta-analysis was conducted to evaluate the effective methods for the early diagnosis of PD using MRS in GP and SN regions so as to provide more precise evidence for clinical diagnosis.

## Materials and Methods

### Search Strategy and Selection Criteria

The Preferred Reporting Items for Systematic Reviews and Meta-analyses protocol and the recommendations of the Cochrane Collaboration were followed in this study for the roles, guidelines, and criteria (19). PubMed, Embase, Web of Science, and Chinese National Knowledge Infrastructure were comprehensively searched for relevant studies till November 2018 by two authors. The search was conducted using the following keywords: “proton magnetic resonance spectroscopy,” “Parkinson disease” or “Parkinson's disease,” and “substantia nigra” or “globus pallidus.” Reference lists of relevant reviews were searched manually. No language restriction was applied. Any discrepancy was resolved by consensus or discussion with a third author when necessary. This search was limited to humans and clinical trials. Studies with unclear data, letters, editorials, and case reports were excluded.

Finally, the selection criteria were as follows: (1) studies that investigated the value of metabolic changes in MRS of SN and GP during the early stages of PD; (2) the most recent publication chosen when data were presented in more than one publication, and the PD data of the same cases from different periods and different literature were included in this study; (3) studies with patients with early-stage PD according to the diagnostic criteria of PD, which was established by the first National Committee for the Extrapyramidal System (20), such as the Hoehn–Yahr stage 1 and 2 or the Unified Parkinson Disease Rating Scale (UPDRS) score <22.6 (21); (4) studies comparing patients with PD vs. other healthy control (HC) groups, and the ipsilateral and contralateral regions recorded in MRS; and (5) studies with metabolic changes in the NAA/Cr in GP and SN.

### Data Extraction

Data from the references were extracted independently by two radiologists *via* a standardized strategy. The two reviewers independently assessed the risk of bias of the included studies. The publication information, such as name of the first author, year of publication, regions of interest (ROIs), voxel size, imaging method, magnetic field, number of patients with PD, number of HC groups, NAA/Cr of the brain in GP and SN, comparative method, and main results vs. control, were collected using standard data extraction forms (Table 1). For different periods of the disease and different neurodegenerative diseases, the first data of early-stage PD were collected and the follow-up data from the publications were omitted according to the third selection criteria. Also, data from other comparative studies using diffusion tensor imaging and susceptibility-weighted imaging were excluded. Any disagreement was resolved by reaching a consensus or consulting a third reviewer.

**Table 1:** Characteristics of included studies.

**References**	**ROIs**	**Voxel size**	**Imaging method**	**Magnetic field**	**PD**	**NAA/Cr ratio**	**HC**	**NAA/Cr ratio**	**Comparative method**	**Main results**
Federico et (1)	Lentiform nucleus	3.4 mL	NA	1.5 T Magnetom Siemens	12	1.82 ± 0.83	10	1.93 ± 0.50	MSA and PSP vs. controls; MSA and PSP vs. PD	A significant decrease; no significant difference
Choe et (17)	Putamen–globus pallidus	1.5 ×1.5 ×1.5 cm^3^ (3.375 mL)	A single voxel technique	1.5 T GE Signa Advantage	7	1.18 ± 0.29	7	1.67 ± 0.27	Symptomatic vs. asymptomatic sides	Significant lateral effect of the NAA/Cr
	Substantia nigra				8	1.55 ± 0.23	8	1.74 ± 0.50		
Federico et (22)	Lentiform nucleus	3.4–8 mL	NA	1.5 T Magnetom Siemens	19	1.65 ± 0.41	12	1.86 ± 0.29	Patients with PD vs. controls	NAA/Cr peak ratio showed a slight but significant decrease
Abe et (15)	Putamen	1 ×1 ×1 cm^3^ (1 mL)	NA	1.5 T GE Signa Advantage	23	1.5 ± 0.2	20	2.2 ± 0.2	PSP, CBD, MSA, and PD, but not VP vs. controls	Significant decrease in the NAA/Cr
Clarke et (23)	Lentiform nucleus	2.0 ×1.75 ×1.75 cm^3^	A single spectrum from a voxel	1.5 T GE Signa Advantage	6	1.31± 0.11	6	1.30 ± 0.24	PD vs. controls	A decrease in the NAA/Cho and an increase in the Cho/Cr
Groger et (18)	Substantia nigra	0.252 mL	Proton 3D-MRSI spectra	3 T Magnetom Siemens	9	2.45 ± 1.55	8	3.34 ± 1.23	Rostral-to-caudal ratios of the metabolites in patients with PD vs. controls	Significant differences (decreased to increased)
Groger et (24)	Substantia nigra	0.252 mL	Proton 3D-MRSI spectra	3 T Magnetom Siemens	20	1.97 ± 1.24	22	2.56 ± 0.73	PD vs. controls;	Significant differences in the rostral-to-caudal NAA/Cr; the rostral NAA/Cr was greater than caudal
Nie et (14)	Basal ganglia	2 ×2 ×2 cm^3^	NA	GE Signa Excite 3.0 T	70	1.81 ± 0.27	74	1.78 ± 0.23	Patients with PD patients (PD-CN) vs. healthy controls	No significant difference
	Substantia nigra				70	1.75 ± 0.30	74	1.80 ± 0.27		
Zhou et (25)	Substantia nigra	7 ×7 ×7 mm^3^	NA	Achieva 3.0 T Philips	30	2.14 ± 1.385	30	1.76 ± 1.203	Ipsilateral and contralateral sides of the affected extremity of patients with PD vs. healthy controls	Significant differences
Wang et (26)	Basal ganglia area	0.47 ×0.63 ×2 mm^3^	NA	1.5 T GE HD propeller	20	1.489 ± 0.113	20	1.932 ± 0.136	Early-stage PD vs. control	NAA/Cr value was highest in the control group
Seraji et a. (27)	Substantia nigra	10 ×10 ×20 mm^3^	16 x 16 x 1 voxels	Siemens 3 T Verio System	23	1.90 ± 0.04	6	2.18 ± 0.09	Compared with baseline values in the PD and control groups	Significantly decreased
Huang and Wang (28)	Striatum	NA	NA	NA	50	1.87 ± 1.88	50	2.45 ± 1.88	PD vs. controls	NAA/Cr was lower than that in controls
	Substantia nigra				50	1.58 ± 1.33	50	2.05 ± 1.23		
Wu et (21)	Globus pallidus	2 ×2 ×1.5 cm^3^	NA	3 T Magnetom Verio, Siemens	14	1.30 ± 0.46	14	1.91 ± 0.23	NAA/Cr for the initially symptomatic side divided by the NAA/Cr for the contralateral side vs. the bilateral	Significantly lower
	Substantia nigra				14	1.69 ± 0.70	14	2.22 ± 0.10		
Zheng et (29)	Basal ganglia	NA	NA	1.5 T GE HD propeller	25	1.485 ± 0.122	25	1.966 ± 0.133	PD vs. controls	Early-PD group showed a downward trend
Jiang et (30)	Substantia nigra	NA	Two-dimensional multivoxel	3.0 T GE Signal HDx	9	2.126 ± 0.465	34	1.909 ± 0.338	PD vs. controls	Not be the diagnostic criteria of PD, but significantly different
Chen and Xu (31)	Striatum	NA	Two- dimensional multivoxel	GE-HDx 1.5 T	20	1.15 ± 0.12	40	1.39 ± 0.21	PD vs. controls	A decrease in NAA/Cr values; statistically significant differences

*CBD, Corticobasal degeneration; MSA, multiple systemic atrophy; PSP, progressive supranuclear palsy; VP, vascular parkinsonism*.

### Statistical Analysis

Continuous outcome variables were measured using mean differences (MDs) and corresponding 95% confidence intervals (CIs). Heterogeneity between studies was detected using Cochrane's Q-test with *P* < 0.05 as a significance level, and then was quantitatively measured through *I*^2^ statistics. Heterogeneity was considered statistically significant when *P* < 0.05 or *I*^2^ >50% according to the Cochrane Handbook for Systematic Reviews of Interventions. A fixed-effects model was used to perform the meta-analysis if the *P*-value of Cochrane's *Q*-tests was >0.05; otherwise, a random-effects model was used. All the data analyses were accomplished using RevMan 5.3 software. A subgroup analysis was conducted to reduce heterogeneity.

Furthermore, the trial sequential analysis (TSA) depended on the quantification of the required information size (RIS), that is, optimal information size (32). TSA was done using TSA 0.9.5.10 Beta software if the number of included trials was more than five. The RIS was estimated using the relative risk reduction and heterogeneity-adjusted information size for continuous outcomes. The result was confirmed as true positive if the cumulative *Z*-curve surpassed the Lan–DeMets trial sequential monitoring boundary or reached the RIS above the conventional significance level line (*Z* = 1.96). This monitoring boundary was used to determine whether the evidence in the present meta-analysis was reliable and conclusive. TSA-adjusted 95% CIs were also presented.

## Results

### Characteristics of Included Studies

**Figure 1:**
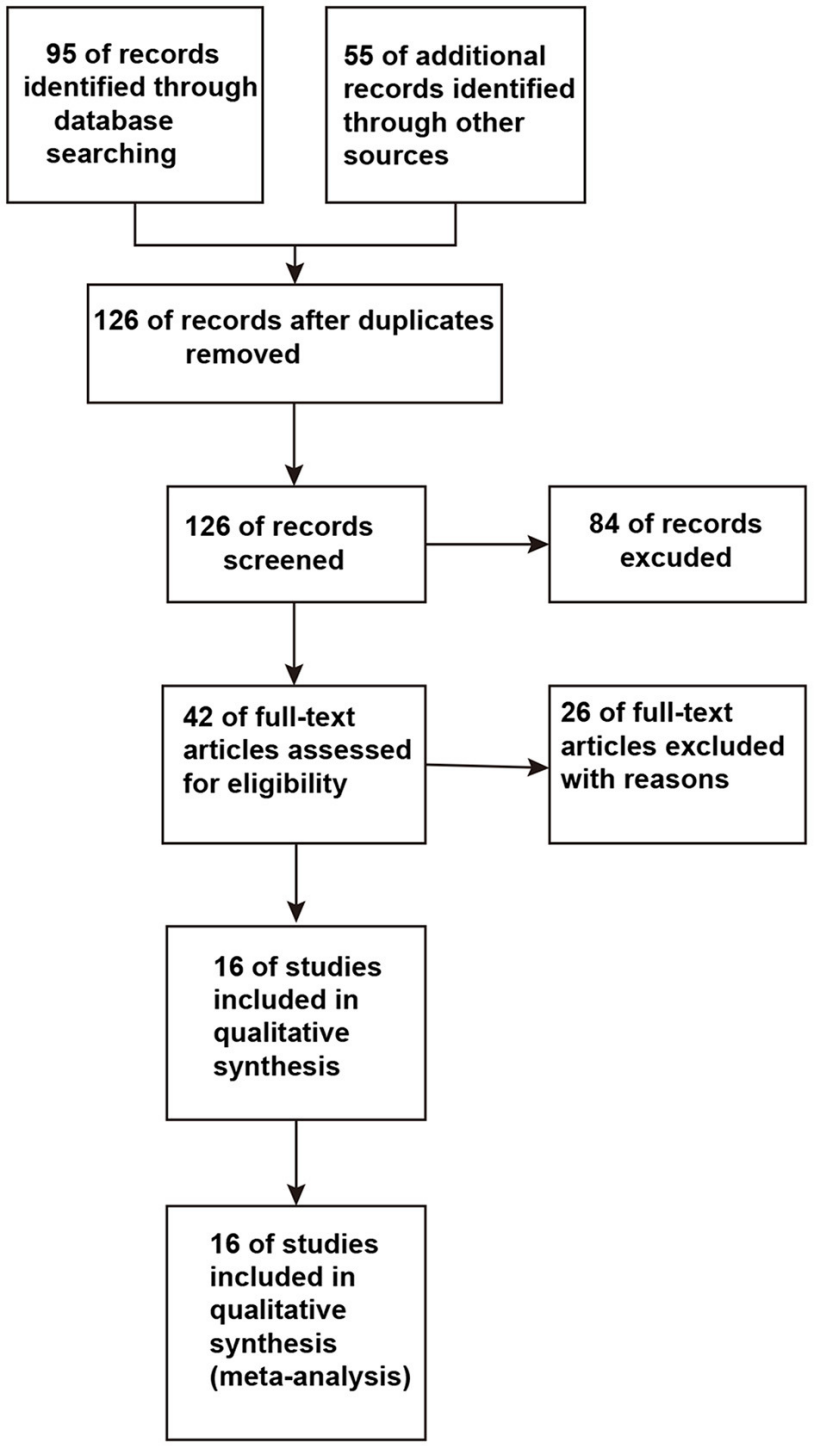
Flowchart of the study selection process.

A total of 126 studies were retrieved from the initial database search. After strict screening according to the eligibility criteria, 16 studies (13–15, 17, 18, 21–31) were finally included in the present meta-analysis. The study selection process is presented in Figure 1. The characteristics of the included studies are shown in Table 1. These studies were published between 1997 and 2018. The sample size of the included studies ranged from 6 to 70 (the total number was 499). Three studies (17, 21, 25) focused on self-control regions of the affected extremity, and two studies (18, 24) included NAA/Cr changes in the rostral and caudal SN regions. The former studies compared the ipsilateral with the contralateral NAA/Cr, while the latter studies collected the data from rostral SN regions like any other studies. Using the aforementioned method, the studies that confounded the results of the overall analysis were avoided for studying relevant outcomes. The comparison of ipsilateral and contralateral regions in the self-control studies was used in the subgroup analysis in this meta - analysis.

### Meta-Analysis

**Figure 2:**
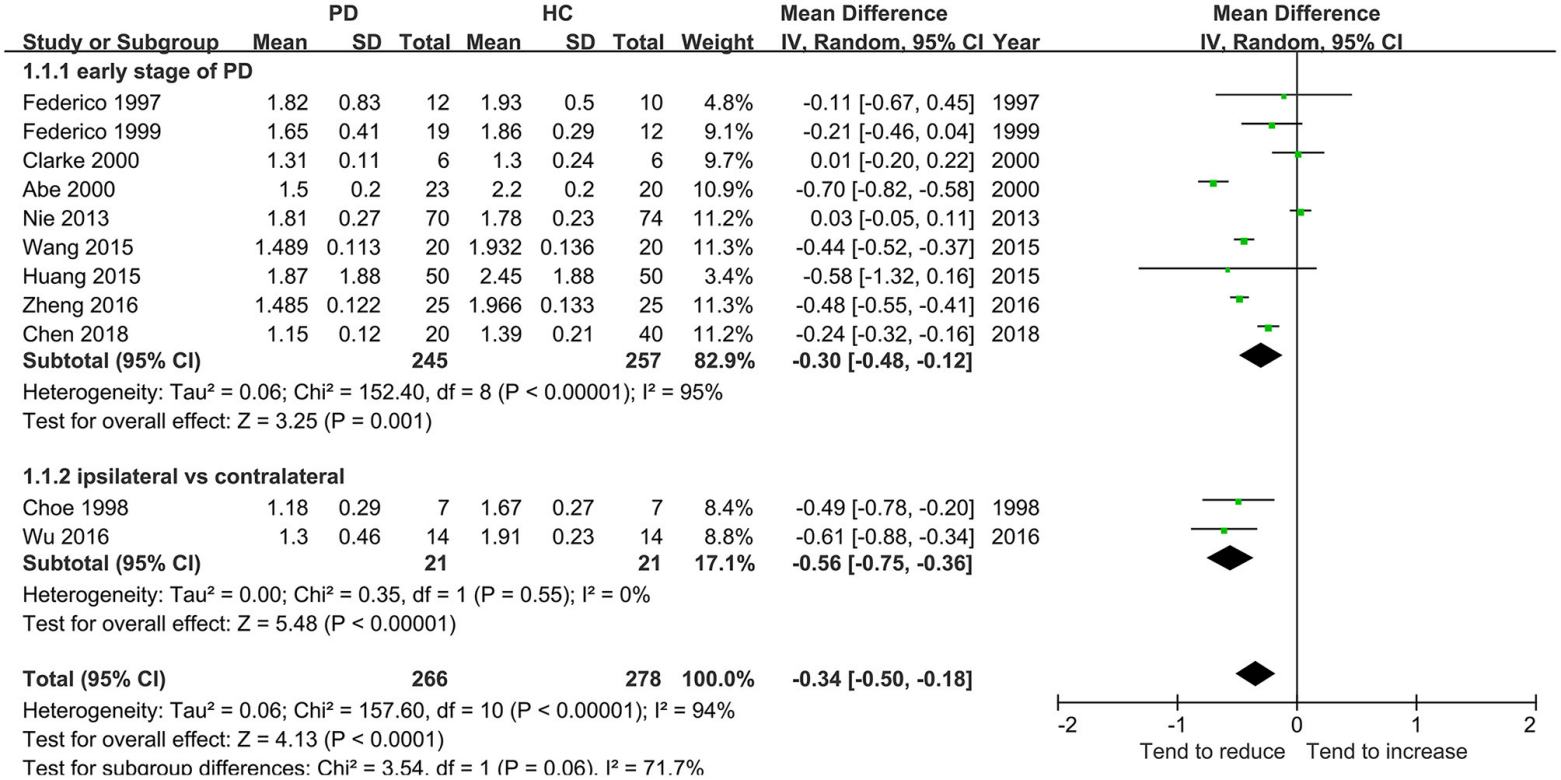
Forest plot of the NAA-to-Cr ratio in GP in patients with early-stage PD vs. HC.

**Figure 3:**
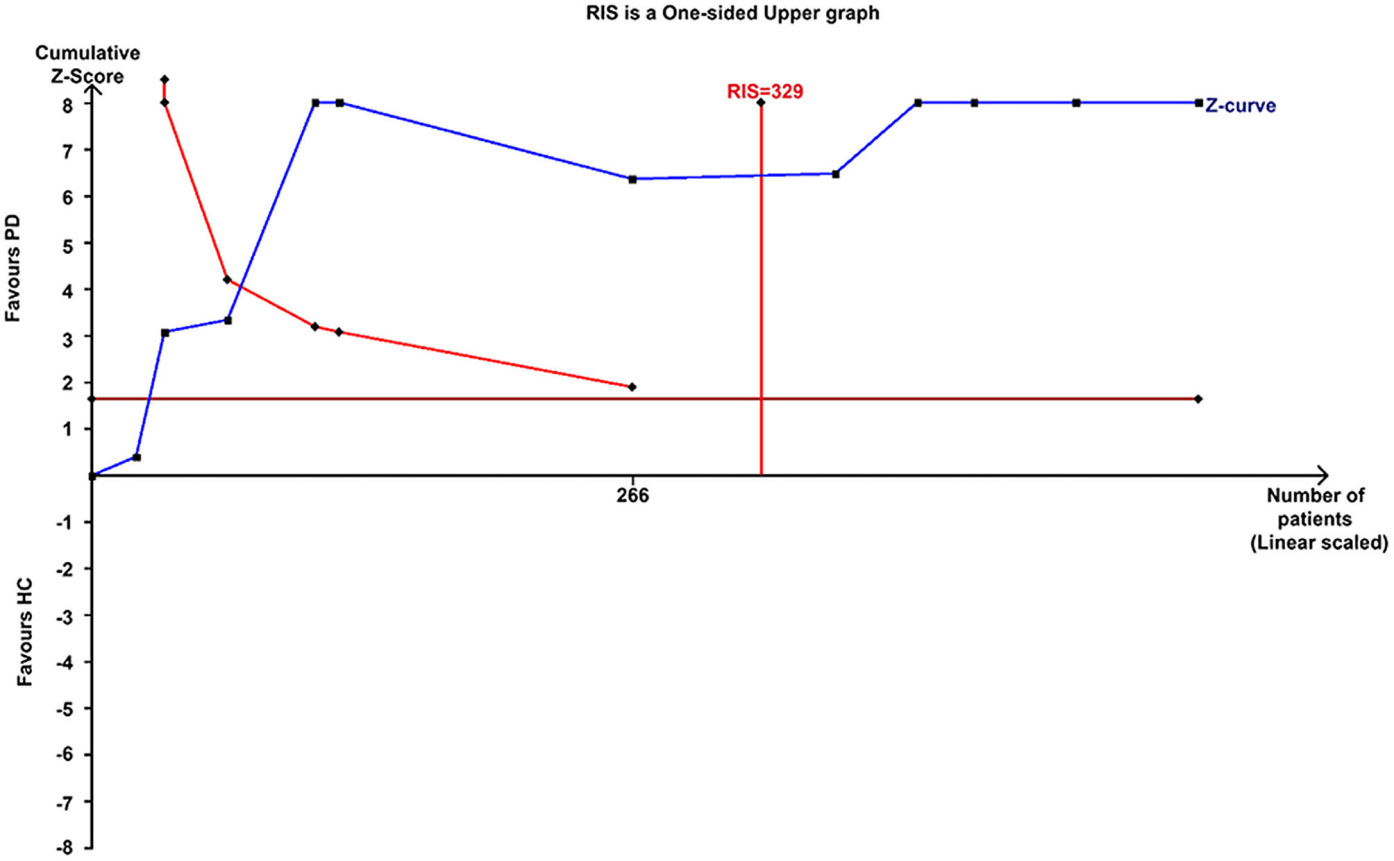
Trial sequential analysis of the NAA-to-Cr ratio in GP.

This meta-analysis involved 11 studies (266 patients with PD) with a significant decrease in the NAA/Cr in the GP of patients with early-stage PD using the random-effects model (MD = −0.34, 95% CI = −0.50 to −0.18; *P* < 0.0001, Figure 2). Moderate-to-high between-study heterogeneity was detected (*P* < 0.00001, *I*^2^ = 94%). The TSA-adjusted 95% CI ranged from −0.37 to −0.30. The TSA results showed that 266 (80.85%) of the RIS of 329 patients was accrued. The cumulative Z-curve crossed the conventional boundary and the trial sequential monitoring boundary for the benefit (Figure 3), indicating that the firm evidence of patients with early-stage PD with the decreasing NAA/Cr in GP was obtained, and the amount of information size accumulated far exceeded than the RIS.

**Figure 4:**
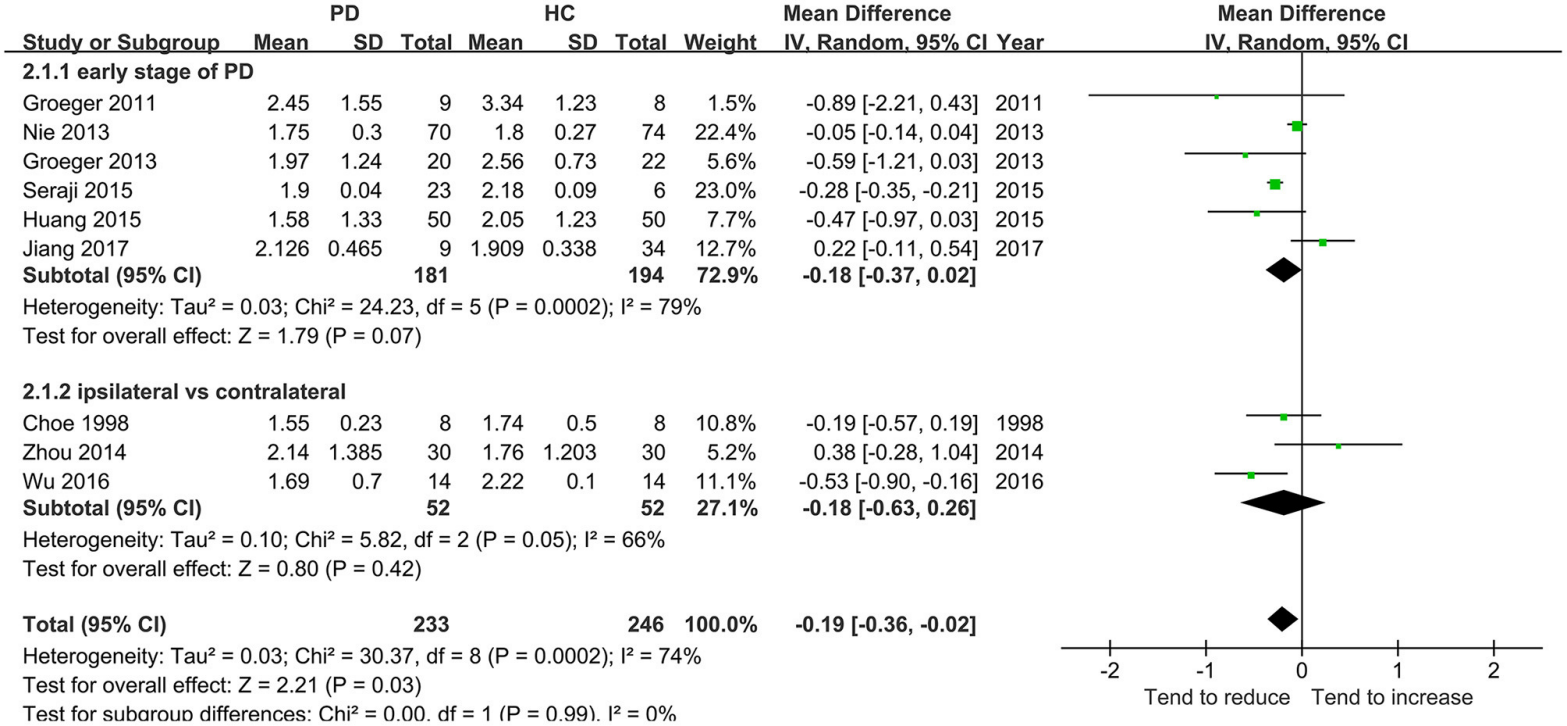
Forest plot of the NAA-to-Cr ratio in SN in early PD vs. HC.

**Figure 5:**
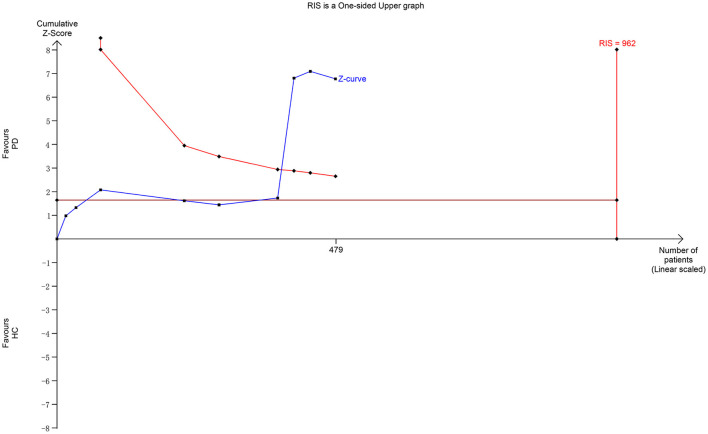
Trial sequential analysis of the NAA-to-Cr ratio in SN.

Nine studies including 233 patients reported the NAA/Cr data in the SN of patients with early-stage PD. The meta-analysis of these studies indicated a significant decrease in the NAA/Cr in the SN of patients with early-stage PD using the random-effects model (MD = −0.19, 95% CI = −0.36 to −0.02; *P* = 0.03). However, the values showed no significant difference in the subgroup of the NAA/Cr that compared patients with PD with HCs or the ipsilateral side with the contralateral side (*P* = 0.07 or 0.42, Figure 4). Moderate-to-high between-study heterogeneity was detected (*P* = 0.0002, *I*^2^ = 74%). The TSA-adjusted 95% CI ranged from −0.24 to −0.13. The TSA result showed that 479 (49.79%) of the RIS of 962 patients was accrued. The cumulative *Z*-curve crossed the conventional boundary and the trial sequential monitoring boundary for the benefit (Figure 5). This finding indicated that the firm evidence of the patients with early-stage PD having a decreasing NAA/Cr in SN was obtained, although the accumulated information size fell short of the RIS.

## Discussion

In the present meta-analysis, a total of 16 studies were reviewed for the early diagnosis of PD with ^1^H-MRS over the past few decades. This meta-analysis suggested that the decreased NAA/Cr indicated the early neuronal loss in the GP or SN of patients with PD patients detected using MRS.

The analysis of the first 11 studies including 266 patients with early-stage PD showed that the NAA/Cr in GP was significantly associated with a decreasing tendency (whether in total or in a subgroup, as shown in Figure 2). This was confirmed by TSA, where the cumulative *Z*-curve of the NAA/Cr surpassed the trial sequential monitory boundary. A comparison with the standard statistical analysis of meta-analysis showed that the results of TSA could adjust the false positives or false negatives. The accumulated information size far exceeded the RIS. Therefore, it was concluded that no more experiments were required for confirmation. The significant difference was so high that the conclusion differed from the finding of a previous study (33) in which no significant changes in the NAA/Cr were observed in the basal ganglia. Although the study (13, 14) showed no significant differences in the NAA/Cr in the SN of patients with PD, a considerable decrease in the NAA/Cr was observed.

The diagnosis of PD currently relies on the development of certain syndromes evaluated using Hoehn-Yahr or UPDRS scores (34). However, these syndromes appear only years after the loss of dopaminergic neurons when the 50–60% of the neuromelanin concentration is lost in SN (35). The present meta-analysis deduced back and forth by retrospective analysis showed a significantly subtle decreasing tendency of the NAA/Cr in the SN of patients with early-stage PD. However, no significant difference in the NAA/Cr in SN was observed in each subgroup of symptomatic vs. asymptomatic sides or early-stage PD vs. HCs. The result of TSA showed that the cumulative *Z*-curve of the NAA/Cr surpassed the trial sequential monitory boundary, and the accumulated information size had fallen short of RIS. The result was not reliable. For example, the study by Nie et al. (14) showed no significant differences in SN and basal ganglia. Also, it was argued that the NAA/Cr declined in patients with PD in 3 months, and might act as a reliable marker of dopaminergic neuronal viability (27). Hence, more studies should be conducted to prove a significant decrease in the NAA/Cr in SN during the early stage of PD.

This study had some limitations. First, the analysis was based on published results using different methods, magnetic fields, voxel sizes, and ROIs, leading to measurement errors. Therefore, the quality of the included studies was relatively low. As shown in Table 1, although most of the studies adequately reported the machine protocols, several domains still showed “unclear” results due to insufficient information obtained from the studies. Second, the studies about other metabolisms, such as NAA, Cho, and γ-aminobutyric acid, could not be included owing to the limited search of related studies, inevitably influencing the precision of the database analysis. Furthermore, the gray literature was not collected, although the databases were searched more comprehensively. Third, the significant heterogeneity might have influenced the validity of the meta-analysis. The heterogeneity might have derived from different areas of the brain as well as from different stages of the disease. Ultimately, the sample size of the present meta-analysis was not large enough. For the outcome of SN with NAA/Cr, 49.79% of the RIS was accrued. Therefore, further studies are warranted to verify these findings.

## Conclusions

This meta-analysis found that the decrease in the NAA/Cr in GP and SN was significant during the early stage of PD. These observations suggest that the decrease in the NAA/Cr in GP and SN acts as a better supplement or adjunct for a diagnostic marker in patients with early-stage or suspected ones of ataxia or parkinsonism. Large sample size and high-quality studies are needed to further evaluate the effect of the decrease in the NAA/Cr in SN on the diagnosis of patients with early-stage PD. However, it is reasonable to say that MRS may serve as an effective clinical tool for decision-making and effectively preventing the progression of PD.

## Data Availability Statement

The original contributions presented in the study are included in the article/Supplementary Material, further inquiries can be directed to the corresponding author/s.

## Author Contributions

WG, CH, and JC: guarantors of the integrity of the entire study. JL and CH: study concepts, design, and statistical analysis. JL and WG: literature research. JL, CH, and JC: manuscript preparation. WG, JL, CH, and JC: manuscript editing. All authors read and approved the final manuscript.

## Funding

This study was funded by the Changshu Bureau of Science and Technology Project (CR201723 and CS202021) and the Sanitation and Health Committee Significant Project of Changshu (csws201824).

## Conflict of Interest

The authors declare that the research was conducted in the absence of any commercial or financial relationships that could be construed as a potential conflict of interest.

## Publisher's Note

All claims expressed in this article are solely those of the authors and do not necessarily represent those of their affiliated organizations, or those of the publisher, the editors and the reviewers. Any product that may be evaluated in this article, or claim that may be made by its manufacturer, is not guaranteed or endorsed by the publisher.
