# Supplementary material for: Proton Magnetic Resonance Spectroscopy for the Early Diagnosis of Parkinson Disease in the Substantia Nigra and Globus Pallidus: A Meta-Analysis With Trial Sequential Analysis
Source: Front Neurol. 2022 Jun 16;13:838230. doi: 10.3389/fneur.2022.838230 (PMC9244590; doi:10.3389/fneur.2022.838230)
Supplement: Supplementary file 1 [file Data_Sheet_1.docx]

***Supplementary Material for Manuscript***

## Supplementary Tables

**Table S1.** Original data table of selected literatures about NAA/Cr in basal ganglia including lentiform nucleus which is made up of the putamen and GP, caudate, and thalamus.

| Author and Year | ROIs | PD | | Healthy Control | |
| --- | --- | --- | --- | --- | --- |
|  |  | NAA/Cr | No. | NAA/Cr | No. |
| Davie 1995 | lentiform nucleus | 1.19-2.31 1.82 | 9 | 1.61-2.0 1.76 | 9 |
| Federico 1997 | lentiform nucleus | 1.82±0.83 | 12 | 1.93±0.50 | 10 |
| Choe 1998 | putamen | 1.18±0.29 | 7 | 1.67±0.27 | 7 |
|  | putamen | 1.22±0.28 | 8 | 1.58±0.38 | 8 |
| Federico 1999 | lentiform nucleus | 1.65±0.41 | 19 | 1.86±0.29 | 12 |
| Taylor 1999 | striatum | 1.18-3.68 1.94 | 12 | 1.23-3.09 2.07 | 12 |
| Abe 2000 | putamen | 1.5±0.2 | 23 | 2.2±0.2 | 20 |
| Clarke 2000 | lentiform nucleus | 1.31±0.11 | 6 | 1.30±0.24 | 6 |
| Lucetti 2001 | basal ganglia | 1.21±0.12 | 10 | 1.34±0.11 | 10 |
| Zheng 2004 | lentiform nucleus | 27.32±8.73/13.64±4.21 | 25 | 24.36±7.47/13.89±4.87 | 25 |
| Hattingen 2009 | putamen(HYI-II) | 13.92±1.94/8.51±1.48 | 15 | 13±1.7/8.19±1.26 | 15 |
|  | putamen(HYIII-IX) | 12.38±1.61/8.41±1.69 | 8 | 12.37±0.89/8.39±0.74 | 8 |
| Guevara 2010 | lentiform nucleus | 6.62±0.8 | 11 | 6.77±1.2 | 18 |
|  | putamen | 6.88±1.2 | 11 | 7.01±1.6 | 18 |
| Nie 2013 | basal ganglia (PD-CN) | 1.81±0.27 | 70 | 1.78±0.23 | 74 |
|  | basal ganglia (PD-MCI) | 1.78±0.27 | 66 |  |  |
| Wu 2016 | Globus Pallidus | 1.22±0.45 | 30 | 1.74±0.19 | 30 |
| Guan 2017 | Globus pallidus | 1.44-1.93 1.65 | 42 | 1.90-3.48 2.84 | 20 |
| Huang 2015 | striatum(L) | 1.87±1.88 | 50 | 2.45±1.88 | 50 |
|  | striatum(R) | 1.89±2.11 | 50 | 2.48±2.01 | 50 |
| Wang 2015 | basal ganglia (PD-MCI) | 1.489±0.113 | 20 | 1.932±0.136 | 20 |
|  | basal ganglia (PD-CN) | 0.952±0.081 | 20 |  |  |
| Chen 2018 | striatum (PD-MCI) | 1.15±0.12 | 20 | 1.39±0.21 | 40 |
|  | striatum (PD-CN) | 0.75±0.15 | 20 |  |  |
| Zheng 2016 | basal ganglia (PD-MCI) | 1.485±0.122 | 25 | 1.966±0.133 | 25 |
|  | basal ganglia (PD-CN) | 0.932±0.087 | 25 |  |  |

**Table S2.** Original data table of selected literatures about NAA/Cr in substantia nigra.

| Author and Year | ROIs | PD | Healthy Control | | | |
| --- | --- | --- | --- | --- | --- | --- |
|  |  | NAA/Cr | | No. | NAA/Cr | No. |
| Choe 1998 | Substantia Nigra (L) | 1.20±0.36 | | 7 | 1.99±0.55 | 7 |
|  | Substantia Nigra (R) | 1.55±0.23 | | 8 | 1.74±0.50 | 8 |
| Hattingen 2009 | Midbrain (HYI-II) | 12.91±2.37/6.75±1.54 | | 15 | 13.16±2.15/6.91±1.05 | 14 |
|  | Midbrain (HYIII-IX) | 11.06±2.31/5.68±1.13 | | 9 | 11.52±1.75/5.71±1.45 | 10 |
| Groeger 2011 | Substantia Nigra(rostral) | 2.45±1.55 | | 9 | 3.34±1.23 | 8 |
|  | Substantia Nigra(caudal) | 4.92±2.96 | | 9 | 2.03±0.67 | 8 |
| Groeger 2013 | Substantia Nigra(rostral) | 1.97±1.24 | | 20 | 2.56±0.73 | 22 |
|  | Substantia Nigra(caudal) | 3.47±2.37 | | 20 | 1.85±0.51 | 22 |
| Nie 2013 | Substantia Nigra (PD-CN) | 1.75±0.30 | | 70 | 1.80±0.27 | 74 |
|  | Substantia Nigra (PD-MCI) | 1.74±0.27 | | 66 |  |  |
| Zhou 2014 | Substantia Nigra | 2.14±1.385 | | 30 | 1.76±1.203 | 30 |
| Seraji 2015 | Substantia Nigra | 1.90±0.04 | | 23 | 2.18±0.09 | 6 |
| Wu 2016 | Substantia Nigra | 1.32±0.66 | | 30 | 1.74±0.12 | 30 |
| Guan 2017 | Substantia Nigra | 1.40-2.10 1.80 | | 42 | 2.27-3.26 2.57 | 20 |
| Huang 2015 | Substantia Nigra (L) | 1.58±1.33 | | 50 | 2.05±1.23 | 50 |
|  | Substantia Nigra (R) | 1.62±1.23 | | 50 | 2.06±1.35 | 50 |
| Jiang 2017 | Substantia Nigra (PD-I) | 2.279±0.435 | | 9 | 2.079±0.381 | 34 |
|  | Substantia Nigra (PD-II) | 2.191±0.953 | | 10 |  |  |
|  | Substantia Nigra (PD-III) | 2.024±0.585 | | 12 |  |  |
|  | Substantia Nigra (PD-IL) | 2.126±0.465 | | 9 | 1.909±0.338 | 34 |
|  | Substantia Nigra (PD-IIL) | 2.117±0.634 | | 10 |  |  |
|  | Substantia Nigra (PD-IIIL) | 2.100±0.686 | | 12 |  |  |

**Table S3.** Data for induction, summary and transformation about NAA/Cr in globus pallidus with PD patients and healthy control.

| References For GP | PD | | | Healthy Control | | |
| --- | --- | --- | --- | --- | --- | --- |
|  | Mean | SD | Total | Mean | SD | Total |
| Federico 1997 | 1.82 | 0.83 | 12 | 1.93 | 0.50 | 10 |
| Choe 1998 | 1.18 | 0.29 | 7 | 1.67 | 0.27 | 7 |
| Federico 1999 | 1.65 | 0.41 | 19 | 1.86 | 0.29 | 12 |
| Abe 2000 | 1.5 | 0.2 | 23 | 2.2 | 0.2 | 20 |
| Clarke 2000 | 1.31 | 0.11 | 6 | 1.30 | 0.24 | 6 |
| Nie 2013 | 1.81 | 0.27 | 70 | 1.78 | 0.23 | 74 |
| Wu 2016 | 1.30 | 0.46 | 14 | 1.91 | 0.23 | 14 |
| Huang 2015 | 1.87 | 1.88 | 50 | 2.45 | 1.88 | 50 |
| Wang 2015 | 1.489 | 0.113 | 20 | 1.932 | 0.136 | 20 |
| Zheng 2016 | 1.485 | 0.122 | 25 | 1.966 | 0.133 | 25 |
| Chen 2018 | 1.15 | 0.12 | 20 | 1.39 | 0.21 | 40 |

**Table S4.** Data for induction, summary and transformation in substantia nigra with PD patients and healthy control.

| References For SN | PD | | | Healthy Control | | |
| --- | --- | --- | --- | --- | --- | --- |
|  | Mean | SD | Total | Mean | SD | Total |
| Choe 1998 | 1.55 | 0.23 | 8 | 1.74 | 0.5 | 8 |
| Groeger 2011 | 2.45 | 1.55 | 9 | 3.34 | 1.23 | 8 |
| Groeger 2013 | 1.97 | 1.24 | 20 | 2.56 | 0.73 | 22 |
| Nie 2013 | 1.75 | 0.30 | 70 | 1.80 | 0.27 | 74 |
| Zhou 2014 | 2.14 | 1.385 | 30 | 1.76 | 1.203 | 30 |
| Seraji 2015 | 1.90 | 0.04 | 23 | 2.18 | 0.09 | 6 |
| Wu 2016 | 1.69 | 0.70 | 14 | 2.22 | 0.10 | 14 |
| Huang 2015 | 1.58 | 1.33 | 50 | 2.05 | 1.23 | 50 |
| Jiang 2017 | 2.126 | 0.465 | 9 | 1.909 | 0.338 | 34 |
